# The effect of oral care education and reminder cards on patients receiving hemodialysis treatment: a quasi-experimental study

**DOI:** 10.1186/s12903-026-09310-6

**Published:** 2026-07-15

**Authors:** Sevda Ates, Ceyda Cetinkayalı

**Affiliations:** Department of Nursing, Faculty of Health Sciences, Canakkale Onsekiz Mart University, Canakkale, Turkey

**Keywords:** Hemodialysis, Oral health, Oral care, Adherence, Nursing

## Abstract

**Background:**

Preserving dental health in patients undergoing hemodialysis is crucial for enhancing quality of life. Nonetheless, interventions pertaining to oral health have not been adequately studied. The aim is to investigate the impact of oral care education and reminder cards concerning oral health on the oral health and illness adaptation of persons undergoing hemodialysis.

**Methods:**

The study employed a quasi-experimental design with a single group, utilising pre-test and post-test measurements. The study included 82 individuals (aged ≥ 18 years) receiving treatment in the hemodialysis units of two different hospitals in Turkey. Sociodemographic and clinical information was obtained from medical records. The “Adaptation to Chronic Illness Scale” and the “Oral Assessment Guide” were employed to assess oral health and illness adaptation. A one-week intervention was conducted, using daily oral health reminder cards following oral care teaching. The Independent Samples T-Test was used for comparing the mean scores of the OAG and ACIS across two categorical variables, and One-Way Analysis of Variance (ANOVA) was used for more than two categorical variables.

**Results:**

The results indicate a significant short-term improvement in oral health and illness adaptation levels one week following the intervention (*p* = 0.00). Specifically, a significant difference was found between the pre-test (15.51 ± 2.67; 84.37 ± 9.14, respectively) and post-test (12.61 ± 3.83; 86.26 ± 9.66, respectively) scores. Oral health and treatment adherence were better after the use of oral care education and reminder cards (*p* = 0.00). Daily tooth brushing frequency was a common factor influencing oral health and chronic disease adherence levels (*p* < 0.05). The mean scores for oral health and illness adaptation were moderately and negatively correlated before and after the intervention (sırasıyla *r*=-0.46, *r*=-0.52; *p* < 0.01).

**Conclusion:**

In conclusion, the use of oral care education and reminder cards appears to be a promising strategy for achieving short-term improvements in oral health and illness adaptation among hemodialysis patients.

**Trial registration:**

This study protocol was retrospectively registered on the trial registry “Clinical trials.gov PRS”. ClinicalTrials.gov ID is NCT07462208. The registration date is March 10, 2026.

**Supplementary Information:**

The online version contains supplementary material available at https://doi.org/10.1186/s12903-026-09310-6.

## Introduction

Hemodialysis (HD) is the predominant therapy for end-stage renal disease [1]. In patients undergoing HD, the progression of the disease and reliance on therapy can complicate illness adaptation [2–5]. The failure of HD patients to comply with fluid restrictions and dietary guidelines can result in adverse complications, including heightened interdialytic weight gain, augmented cardiac workload, congestive heart failure, hypertension, dyspnea, peripheral edema, pulmonary edema, uremia, and ascites. The resultant issues, along with an elevated symptom burden, can adversely impact quality of life [3, 6]. Oral health issues, commonly observed in patients undergoing HD treatment, adversely impact quality of life and present clinically [7–9].

### Literature review

Oral diseases impact over approximately 3.7 billion individuals. Oral health issues remain a considerable health concern, despite their preventability [10]. Patients undergoing HD often exhibit periodontitis and inflammation of the oral mucosa as prevalent oral health issues [11]. Additionally, coated tongue, xerostomia, oral candidiasis, glossitis, gingivitis, aphthous ulcers, uremic fetor, and scrotal tongue are prevalent findings of the mucosal membrane [12]. Malnutrition, dehydration, insufficient oral hygiene [13], compromised salivary gland function [11], elevated uremic toxins in saliva, kussmaul respiration, and fluid restriction [14] can jeopardize oral health. Additionally, the utilization of parathyroid hormone-lowering pharmaceuticals, low educational attainment, insufficient oral hygiene, dental cavities, non-compliance with dietary and medicinal regimens, daily fluid intake, and diminished potassium levels can exacerbate oral health issues [15].

Oral health is a crucial factor influencing quality of life, general health, and well-being. The disease’s impacts, the imperative of complying with HD treatment, and an augmented caregiving burden may result in the neglect of oral hygiene [16, 17]. The occurrence of degradation in the oral mucous membrane often requires a care intervention [18]. Oral illnesses can be a major source of inflammation; thus, early recognition and intervention might avert possible infections [19]. The WHO (2025) highlights the correlation between oral health and chronic diseases, asserting that oral disorders are preventable by a regular dental hygiene regimen [10]. In this context, therapies that preserve oral health are crucial for promoting health concerning chronic conditions. Integrating oral health assessments into standard nursing and midwifery practices can substantially enhance the health of individuals and communities [20].

Oral health care is a crucial aspect of daily hygiene for hospitalized patients, and nurses play a vital role in assisting patients in sustaining an adequate level of oral health [21]. Prompt detection of oral lesions in patients undergoing HD treatment is essential, along with the implementation of regular oral examinations [8]. Prioritizing oral hygiene in the everyday nursing care of these patients is essential, along with developing interventions for factors influencing oral health [22]. Patients should be encouraged to engage in thorough dental care and adhere to personal oral hygiene directives [9]. Literature contains studies assessing oral health in individuals undergoing HD treatment [7–9, 18, 23, 24], and it is established that education on oral care enhances oral health [25, 26]. The impact of oral health therapies remains ambiguous. This study examined the impact of oral care education and oral health reminder cards on oral health levels and adherence to chronic disease management in individuals undergoing HD therapy.

## Materials and methods

### Study design

This study was conducted using a “single-group pretest-posttest design” quasi-experimental design to assess the impact of oral care education and reminder cards on the oral health and illness adaptation levels of patients undergoing HD treatment [27]. Study reported in accordance with TREND Checklist [28]. The study population consisted of 115 individuals who applied for hemodialysis treatment at the hemodialysis unit of a research and teaching hospital.

### Participants and setting

**Fig. 1:**
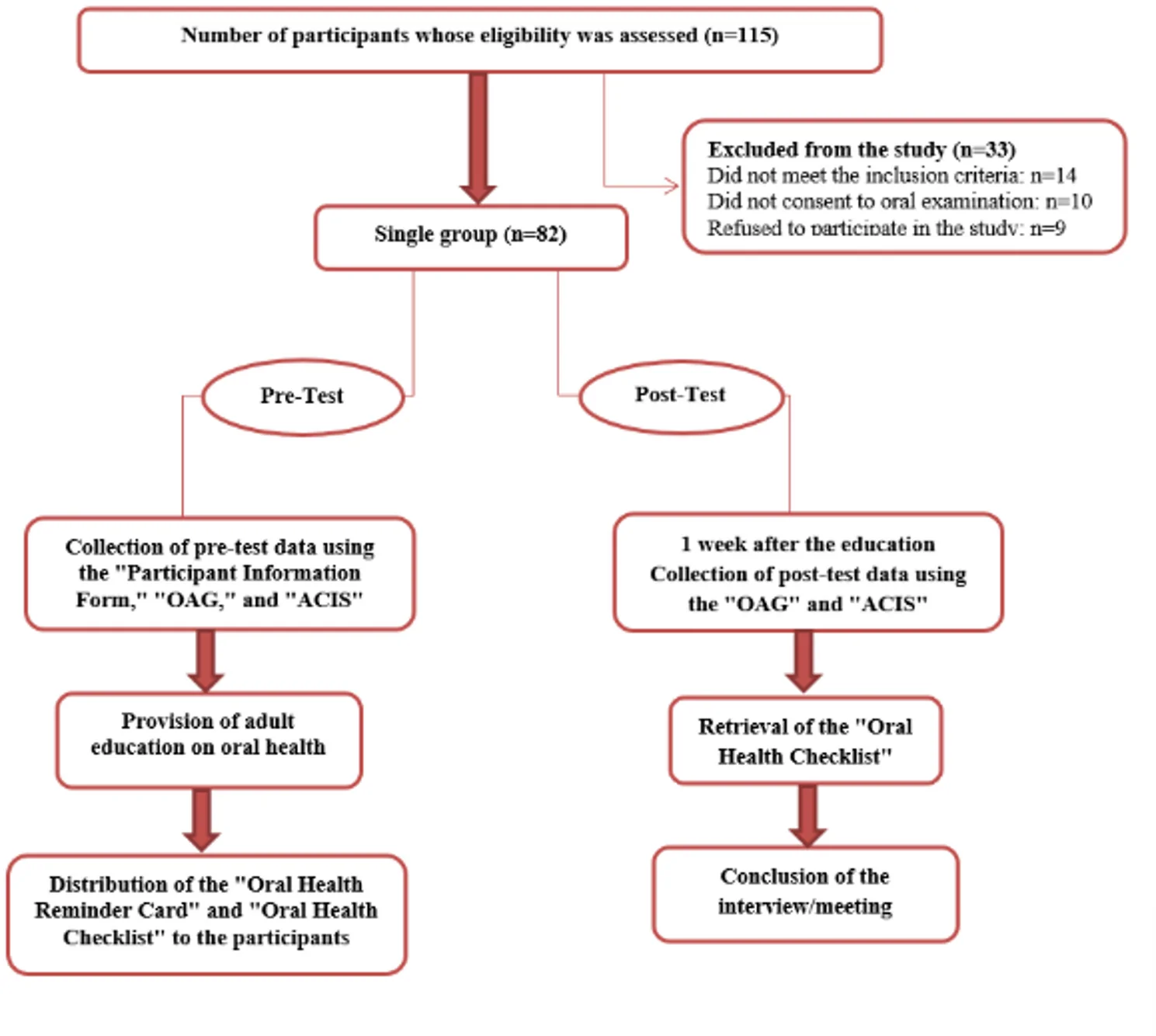
Study flow diagram

The population consisted of 115 individuals who applied to the hemodialysis unit of a research and practice hospital located in a city centre for hemodialysis treatment between July and October 2024. No sample selection was performed, and 82 (71.30%) patients on hemodialysis who met the inclusion criteria of the research were included in the sample. The inclusion criteria were: being 18 years of age or older, receiving HD treatment three or four times a week for at least 6 months, having no hearing, visual, or speech impairment, having no diagnosis of malignancy, and performing self-oral care. Patients who refused to participate in the study (*n* = 9), did not meet the inclusion criteria (*n* = 14), and did not consent to an oral examination (*n* = 10) were not included in the sample (Fig. 1).

### Measures

Patient Information Form: This form, developed from existing research, comprises 17 questions pertaining to the socio-demographic, illness, treatment-related factors, and oral care practices of persons undergoing HD therapy (Supplementary file) [7, 9, 12, 13, 15, 18, 22–24, 29].

Adaptation to Chronic Illness Scale (ACIS): Developed by Atik and Karatepe (2016),10 the scale consists of 25 items and three subdomains, including physical adjustment, social adjustment, and psychological adjustment. The scale is designed as a 5-point Likert scale (with 1 = Strongly Disagree and 5 = Completely Agree). Items 5, 6, 12, 17, 19, 20, 24, and 25 are reverse scored. Maximum score that can be obtained from the scale is 125. Higher scores from the subdomains or the entire scale are indicative of the fact that the patients’ level of adaptation to the disease is also high. The Cronbach’s Alpha value of the scale is 0.88 for the entire scale [30]. In this study, the Cronbach’s alpha value was 0.71 for the pre-test and 0.75 for the post-test.

Oral Assessment Guide (OAG): The guide allows for the assessment of voice, swallowing, lips, tongue, saliva, mucous membrane, gums, and teeth/dentures. The OAG supports the creation of an oral care protocol for patients with impaired oral mucous membranes. Each change related to oral health is scored between 1 and 3 points, and these scores are summed to obtain the total score. Scores obtainable from the OAG range from 8 to 24. A score between 8 and 14 indicates a “risk of impairment in the oral mucous membrane,” while a score between 14 and 24 indicates “impairment in the oral mucous membrane.” According to the OAG, a low score indicates good oral health, whereas high scores suggest poor oral health. A light source and a tongue depressor for each participant were used when evaluating oral health [31].

Oral Health Reminder Card: The card contains a total of 10 items regarding what should be paid attention to in daily oral health and was prepared based on the literature [7, 12, 13, 18, 20, 24, 32, 33]. These cards were produced in a 10 × 15 cm format to ensure portability and visibility. Participants were instructed to place the cards in a prominent location within their homes to serve as a continuous visual cue. Unlike the monitoring checklists, these reminder cards were provided as a permanent resource and were not retrieved from the participants at the end of the study.

Oral Health Checklist: The Oral Health Checklist was developed by the researchers as a clinical monitoring tool, structured in accordance with evidence-based guidelines and existing literature. This form contains the items listed on the Oral Health Reminder Card [7, 12, 13, 18, 20, 24, 32, 33]. Although the checklist was not subjected to formal psychometric validation, its content validity is derived from established oral health protocols for chronic illness management. Participants were asked to implement the items on the checklist and mark them as applied. Participants had the opportunity to evaluate whether they performed the oral health items every day over a one-week period. These completed checklists were collected during the final interview to evaluate individual treatment adherence and behavioral consistency (Supplementary File).

### Interventions

#### Initial Interview and educational intervention

Data were collected between July and October 2024. Before the research data were collected, the research aim the aim of the research, its content, what it covered, and its duration was explained to participant. The data collection form was given by the researchers to the patients who met the inclusion criteria of the study. The participants were asked to answer the questions in the data collection instruments in a way that they thought suitable and to answer all the questions. During the answering period, the researchers were with the individuals and answered the questions asked about the forms. After each survey, the researchers checked the completeness of the questionnaire. If there were omissions in the survey questionnaire, the research subjects were immediately invited to fill in the missing information. Participants provided signed informed consent forms, which were returned with the questionnaires. Data collection took an average of 20 min for each participant and was based on self-report through face-to-face interviews.

The oral health of all participants was assessed by a single researcher using the OAG. Following the pre-test assessment, a standardized 15-minute one-on-one oral health education session was provided. The instructional content included: oral health in adults; the consequences of poor oral hygiene; points to consider for oral health; demonstration of the brushing technique; coping with care-resistant behaviors; and how to use the oral health reminder card and checklist [7, 12, 13, 18, 20, 24, 32, 33]. Following the educational session, each patient received an oral health reminder card and a checklist to monitor their daily oral hygiene routines.

#### Second interview and post-intervention assessment

After a one-week intervention that included the use of daily oral health reminders (card and checklist) following the oral care education, a post-test was administered to the participants using the ACIS and OAG. The checklists maintained by the participants during the home-care period were collected for review. Post-intervention oral health scores were recorded systematically on the data collection forms to evaluate the impact of the educational intervention.

### Data analysis

Data were analyzed in using SPSS version 23 (IBM Corp, Armonk, NY, USA) applying a 95% confidence interval and *p*<0.05. The characteristics of the participants were analyzed using frequency and percentage analyses, and mean and standard deviation statistics were used in scale analyses. It was assumed that the variables showed a normal distribution if their skewness and kurtosis values were within ± 2. The Independent Samples T-Test was used for comparing the mean scores of the OAG and ACIS across two categorical variables, and One-Way Analysis of Variance (ANOVA) was used for more than two categorical variables. Tukey HSD, LSD, and Games-Howell tests were applied as post hoc tests. Pearson and Spearman correlation analyses were used for the correlation between continuous variables [34, 35].

### Ethical considerations

The study complies with the ethical standards specified in the Helsinki Declaration. The study was approved by the University Institutional Review Board (date: 03 June, 2024, No: 2024/06 − 03). Appropriate permission was obtained from the institutions where the study was conducted (No: E-27222899-800-2400117142, No: E-97769597-799-255643896). Informed consent was obtained from all individual participants included in the study. They were given the option to voluntarily withdraw their informed consent at any point during the research and their personal data were kept strictly confidential throughout the study. The full trial protocol and statistical analysis plan can be accessed on the clinical trial registry page at www.clinicaltrials.gov (ID: NCT07462208).

## Results

### Descriptive characteristics of individuals receiving hemodialysis

The mean age of the participants was 56.26 years, the mean duration of HD treatment was 3.91 years, and the mean number of daily medications used was 5.04. The majority of participants were male (53.7%), primary school graduates (48.8%), married (79.3%), had children (81.7%), were retired (45.1%), were not receiving oxygen therapy (91.5%), had an accompanying chronic disease (69.5%), and lived with their families (91.5%). The majority had not received oral care education (93.9%) (Table 1).

**Table 1:** Descriptive characteristics of individuals receiving hemodialysis

Characteristics	Mean ± SD (min-max)	
Age	56.26 ± 16.83 (22.00–91.00)	
Duration of HD treatment (years)	3.91 ± 4.72 (1.00–33.00)	
Total number of medications used (days)	5.04 ± 3.52 (1.00–20.00)	

		*n* (%)
Gender	Female	38 (46.3)
	Male	44 (53.7)
Educational background	Basic literacy	7 (8.5)
	Primary school	40 (48.8)
	High school	21 (25.6)
	Bachelor’s degree	14 (17.1)
Marital status	Married	65 (79.3)
	Single	17 (20.7)
Employment status	Employed	17 (20.7)
	Unemployed	28 (34.1)
	Retired	37 (45.1)
Income status	Income is less than expenses	20 (24.4)
	Income is equal	54 (65.9)
	Income is greater than expenses	8 (9.8)
Person living together	Alone	7 (8.5)
	With family (spouse/child/mother/father)	75 (91.5)
Having a child	Yes	67 (81.7)
	No	15 (18.3)
Smoking status	Yes	15 (18.3)
	No	67 (81.7)
Alcohol use status	Yes	4 (4.9)
	No	78 (95.1)
Oxygen therapy status	Yes	7 (8.5)
	No	75 (91.5)
Chronic disease	Yes	57 (69.5)
	No	25 (30.5)
Status of receiving oral care training	Yes	5 (6.1)
	No	77 (93.9)

### Toothbrushing frequency pre- and post-intervention

At baseline, the proportion of patients who reported brushing their teeth twice daily was 20.7% (*n* = 17), while the proportion of those who never brushed their teeth was 37.8% (*n* = 31). At the one-week follow-up, the rate of patients brushing their teeth twice daily was 72.0% (*n* = 59), and the rate of those who reported never brushing their teeth was 28.0% (*n* = 23) (Table 2).

**Table 2:** Toothbrushing frequency pre- and post-intervention

Toothbrushing Frequency	Pre-test	Post-test
	*n*(%)	*n*(%)
Every other day	19 (23.2)	
Once a day	15 (18.3)	
Twice a day	17 (20.7)	59 (72.0)
Never brushes	31 (37.8)	23 (28.0)

### Oral health level and influencing factors

The mean OAG score of the participants was 15.51. The mean post-education OAG score was lower than the mean pre-education OAG score (*p* = 0.00) (Table 3).

**Table 3:** OAG and ACIS pretest-posttest scores

*N* = 82	Pre-test	Post-test		
	Mean ± SD (min-max)	Mean ± SD (min-max)	T	*p*
OAG	15.51 ± 2.67 (10.00–20.00)	12.61 ± 3.83 (8.00–21.00)	8.700	0.00*
ACIS	84.37 ± 9.14 (61.00-111.00)	86.26 ± 9.66 (62.00-113.00)	-6.386	0.00*
Physical adaptation	38.11 ± 4.73 (25.00–49.00)	38.82 ± 5.38 (25.00–49.00)	-3.403	0.00*
Social adaptation	23.98 ± 4.05 (15.00–34.00)	25.09 ± 3.96 (15.00–34.00)	-7.872	0.00*
Psychological adaptation	22.28 ± 3.40 (15.00–30.00)	22.35 ± 3.33 (15.00–30.00)	-1.284	0.20

*Abbreviation SD * standard deviation, value in bold significant, *T *The Independent Samples T-Test, *p < 0.05; *OAG * Oral Assessment Guide, *ACIS * Adaptation to Chronic Illness Scale

The mean OAG pre-test scores were higher in individuals whose income was less than their expenditure (*p* = 0.04); and in those who never brushed their teeth (*p* = 0.00). The mean OAG post-test scores were higher for those who never brushed their teeth compared to those who brushed twice a day (*p* = 0.00) (Table 4).

### Chronic disease adherence level and influencing factors

The mean ACIS score of the participants was 84.37. The mean post-education ACIS score was higher than the mean pre-education ACIS score (*p* = 0.00) (Table 3). The mean ACIS pre-test scores were higher in those with a high school education or above (*p* = 0.03), in those who brushed their teeth once or twice a day (*p* = 0.02), in those whose income was equal to or greater than their expenditure (*p* = 0.01) and in non-smokers (*p* = 0.04). The mean ACIS post-test scores were low in those who never brushed their teeth (*p* = 0.00), and in those whose income was less than their expenditure (*p* = 0.03) (Table 5).

**Table 4:** Factors affecting OAG

		***n***	**Pre-test**				**Post-test**			
			**Mean ± SD**	**Test**		***p***	**Mean ± SD**	**Test**		***p***
Gender	Female	38	15.68 ± 2.68	T	0.540	0.59	12.24 ± 3.88	T	-0.817	0.42
	Male	44	15.36 ± 2.68				12.93 ± 3.81			
Educational background	Below high school	47	15.85 ± 2.64	T	1.339	0.18	12.85 ± 3.96	T	0.658	0.51
	High school and above	35	15.06 ± 2.68				12.29 ± 3.69			
Marital status	Married	65	15.62 ± 2.69	T	0.683	0.50	12.75 ± 3.82	T	0.663	0.51
	Single	17	15.12 ± 2.62				12.06 ± 3.93			
Employment status	Employed	17	15.35 ± 2.03	F	0.281	0.76	12.82 ± 3.71	F	0.150	0.86
	Unemployed	28	15.82 ± 2.70				12.82 ± 4.18			
	Retired	37	15.35 ± 2.94				12.35 ± 3.70			
Income status	a. Income is less than expenses	20	16.55 ± 2.76	T	2.039	0.04* **(b < a)**	13.55 ± 3.85	T	1.267	0.21
	b. Income is equal to/more than expenses	62	15.18 ± 2.57				12.31 ± 3.81			
Having a child	Yes	67	15.76 ± 2.73	T	1.811	0.07	12.73 ± 3.92	T	0.605	0.55
	No	15	14.40 ± 2.10				12.07 ± 3.47			
Smoking status	Yes	15	15.93 ± 2.63	T	0.674	0.50	13.53 ± 3.68	T	1.033	0.31
	No	67	15.42 ± 2.69				12.40 ± 3.86			
Concomitant chronic disease	Yes	57	15.42 ± 2.72	T	-0.465	0.64	12.23 ± 3.65	T	-1.369	0.18
	No	25	15.72 ± 2.59				13.48 ± 4.17			
Hypertension	Yes	45	15.58 ± 2.73	T	0.244	0.81	12.00 ± 3.54	T	-1.604	0.11
	No	37	15.43 ± 2.62				13.35 ± 4.09			
Diabetes	Yes	28	15.29 ± 2.59	T	-0.551	0.58	12.39 ± 3.30	T	-0.367	0.72
	No	54	15.63 ± 2.72				12.72 ± 4.10			
Heart failure	Yes	11	15.55 ± 3.70	T	0.044	0.97	11.27 ± 2.33	T	-1.248	0.22
	No	71	15.51 ± 2.51				12.82 ± 3.99			
Coronary artery disease	Yes	14	16.07 ± 2.09	T	0.860	0.39	14.36 ± 4.24	T	1.904	0.06
	No	68	15.40 ± 2.77				12.25 ± 3.68			
Daily tooth brushing status	a. Never brushes	31	17.00 ± 2.21	F	9.916	0.00* **(c**,** d < a)**	14.32 ± 4.09	F	5.450	0.00* **(d < a)**
	b. Every other day	19	15.68 ± 2.38				12.89 ± 3.60			
	c. Once a day	15	14.67 ± 2.29				11.27 ± 3.01			
	d. Twice a day	17	13.35 ± 2.45				10.35 ± 2.76			
Tooth brushing status after training	a. Twice a day	59	15.20 ± 2.57	T	-1.698	0.09	10.76 ± 1.99	T	-11.030	0.00* **(a < b)**
	b. No	23	16.30 ± 2.82				17.35 ± 3.31			

*Abbreviation* *SD * standard deviation, value in bold: significant, *T * The Independent Samples T-Test, *F * Independent Sample *p < 0.05, *OAG * Oral Assessment Guide, The total OAG score ranges from 8 to 24, High scores indicate poor oral health

**Table 5:** Factors affecting ACIS

		***n***	**Pre-test**				**Post-test**			
			**Mean ± SD**	**Test**		***p***	**Mean ± SD**	**Test**		***p***
Gender	Female	38	83.68 ± 10.36	T	-0.625	0.53	86.24 ± 10.40	T	-0.017	0.99
	Male	44	84.95 ± 8.02				86.27 ± 9.11			
Educational background	Below high school	47	82.49 ± 9.77	T	-2.205	0.03* **(a < b)**	84.70 ± 10.08	T	-1.707	0.09
	High school and above	35	86.89 ± 7.66				88.34 ± 8.79			
Marital status	Married	65	84.82 ± 8.89	T	0.869	0.39	86.55 ± 9.38	T	0.543	0.59
	Single	17	82.65 ± 10.14				85.12 ± 10.91			
Employment status	Employed	17	85.24 ± 8.06	F	1.278	0.28	86.41 ± 10.01	F	0.807	0.45
	Unemployed	28	82.14 ± 10.98				84.46 ± 10.99			
	Retired	37	85.65 ± 7.93				87.54 ± 8.42			
Income status	a. Income is less than expenses	20	79.85 ± 10.00	T	-2.632	0.01* **(a < b)**	82.10 ± 9.55	T	-2.268	0.03* **(a < b)**
	b. Income is equal to/more than expenses	62	85.82 ± 8.42				87.60 ± 9.39			
Having a child	Yes	67	84.13 ± 9.41	T	-0.482	0.63	86.01 ± 9.77	T	-0.475	0.64
	No	15	85.40 ± 8.07				87.33 ± 9.43			
Smoking status	a. Yes	15	80.07 ± 9.10	T	-2.055	0.04* **(a < b)**	82.07 ± 9.51	T	-1.887	0.06
	b. No	67	85.33 ± 8.94				87.19 ± 9.51			
Concomitant chronic disease	Yes	57	84.93 ± 9.44	T	0.842	0.40	87.18 ± 9.92	T	1.307	0.20
	No	25	83.08 ± 8.47				84.16 ± 8.88			
Hypertension	Yes	45	84.76 ± 10.17	T	0.424	0.67	87.29 ± 10.55	T	1.068	0.29
	No	37	83.89 ± 7.82				85.00 ± 8.44			
Diabetes	Yes	28	83.07 ± 7.65	T	-0.922	0.36	85.18 ± 8.53	T	-0.725	0.47
	No	54	85.04 ± 9.83				86.81 ± 10.23			
Heart failure	Yes	11	87.09 ± 11.53	T	1.063	0.29	89.82 ± 11.31	T	1.320	0.19
	No	71	83.94 ± 8.74				85.70 ± 9.35			
Coronary artery disease	Yes	14	83.64 ± 8.46	T	-0.323	0.75	85.00 ± 9.45	T	-0.532	0.60
	No	68	84.51 ± 9.33				86.51 ± 9.75			
Daily tooth brushing status	a. Never brushes	31	80.68 ± 9.29	F	3.437	0.02* **(c**,** d > a)**	82.10 ± 10.00	F	3.958	0.01* **(c**,** d > a)**
	b. Every other day	19	84.68 ± 9.35				86.74 ± 9.94			
	c. Once a day	15	87.67 ± 5.27				90.60 ± 5.53			
	d. Twice a day	17	87.82 ± 9.44				89.47 ± 9.21			
Tooth brushing status after training	a. Twice a day	59	85.20 ± 9.23	T	1.335	0.19	88.17 ± 9.20	T	3.011	0.00* **(b < a)**
	b. No	23	82.22 ± 8.73				81.35 ± 9.27			

*Abbreviation* *SD * standard deviation, *T * The Independent Samples T-Test, *F * Independent Sample *p < 0.05; value in bold: significant, *ACIS * Adaptation to Chronic Illness Scale, The total ACIS score ranges from 25 to 125, High score speed indicates high compliance

### Relationship between the OAG and ACIS pretest-posttest scores

The mean pre-test and post-test scores of the OAG and ACIS were moderately and negatively correlated (*r*=-0.46; *r*=-0.52, respectively, *p* < 0.01) (Table 6).

**Table 6:** Relationship Between the OAG and ACIS Pretest-Posttest Scores

		**OAG**		**ACIS**	
		**Pre-test**	**Post-test**	**Pre-test**	**Post-test**
ACIS	*r*	-0.46^1^	-0.52^1^	1.00	1.00
	*p*	0.00*	0.00*		
Age	*r*	0.05^1^	0.01^1^	-0.06^1^	-0.07^1^
	*p*	0.67	0.90	0.61	0.56
Duration of HD treatment (years)	*r*	0.01^2^	-0.02^2^	-0.01^2^	0.02^2^
	*p*	0.91	0.86	0.93	0.86
Total number of medications used (days)	*r*	-0.04^2^	-0.05^2^	0.00^2^	0.00^2^
	*p*	0.73	0.64	0.98	0.98

*Abbreviations * *p < 0.01, **p < 0.05; value in bold: significant, *r*^1^Pearson correlation analysis applied, *r*^2^ Spearman correlation, *OAG * Oral Assessment Guide, *ACIS * Adaptation to Chronic Illness Scale

## Discussion

This study presents the results of a one-week intervention that utilized oral health reminder cards after oral care education. The findings of this study demonstrate a statistically significant improvement in oral health and illness adaptation scores following the implementation of oral care education and reminder cards. However, given the quasi-experimental nature of the study and the absence of a control group, these changes should be interpreted with caution as they cannot be exclusively attributed to the intervention alone. Potential confounding factors, such as increased researcher attention or the Hawthorne effect, may have contributed to the observed outcomes. Furthermore, it was determined that HD patients with better oral health had better chronic disease adherence levels. Periodontal diseases can affect inflammation, immune response, and nutritional status in those undergoing dialysis treatment. Therefore, maintaining oral health and treating periodontal disease in dialysis patients can improve quality of life, and prevent various pathological conditions and mortality [36].

Oral health is affected by tooth brushing status and income level [37, 38]. In the study, it was found that oral health was poor among individuals with low income, those who never brushed their teeth, and those who did not brush their teeth after the education. The literature reports that individuals whose income is less than their expenditure and who do not brush their teeth regularly have poorer chronic disease adherence [39, 40]. Bilgiç and Pehlivan (2023) determined that individuals with multiple chronic diseases and those non-adherent to their diet had more difficulty adapting to the disease [41]. In contrast to these findings, this study found that the presence of chronic disease did not affect the levels of oral health and disease adherence. The reason for this difference may be related to the duration, severity, and number of coexisting chronic diseases.

Neşe and Bayram (2024) found that chronic disease adherence was at a better level in those with a higher education level and in non-smokers [42]. Ekici and Çayır Yılmaz (2025) [39] determined that adherence to the disease was better in women, married individuals, those with a high school education or above, those with good economic status, those who were employed, those who had been receiving HD treatment for 6–8 years, and those using a fistula. In this study, chronic disease adherence levels were better in men and married individuals, but they did not show a significant difference. Chronic disease adherence was found to be at a better level in those with a high school education or above, non-smokers, those with good income status, and those with a tooth brushing habit. The findings support the literature.

Oral care is necessary to prevent oral health problems and related complications that may arise [7, 15, 24]. It is known that the oral care provided to patients receiving HD treatment improves impairment in the oral mucous membrane. However, even if oral health improves, complaints of sensitivity and dryness persist [18]. Cotič et al. (2017) reported that the majority of HD patients brush their teeth once or twice a day [23]. Contrary to these findings, this study determined that the number of individuals brushing their teeth once or twice a day was low, and those who never brushed were more numerous. In the oral health evaluation conducted one week after the education, an increase was observed in the number of people brushing their teeth twice a day. The implementation of education alongside the use of reminder cards and checklists was associated with an increase in awareness among HD patients. This increased awareness is thought to have contributed to the reduction in oral care neglect observed during the one-week study period. However, given the short-term follow-up, further research is needed to determine if this heightened awareness leads to sustained behavioral changes in long-term oral hygiene practices.

It has been reported that diabetic individuals with coexisting periodontal disease have poor chronic disease adherence, while those with good oral health have better health-related quality of life [17, 26]. Çevik (2019) determined that individuals who do not go for regular medical check-ups and do not engage in protective oral health behaviors have a poorer oral health-related quality of life [38]. It is known that oral care education given to individuals with chronic diseases positively affects oral health and increases their quality of life [26]. Aşkın (2023) determined that oral health education provided to individuals who have suffered a stroke improved their oral health-related quality of life [25]. Similarly, educational interventions for oral care in diabetic patients positively affected oral health knowledge and disease management [43].

The use of reminders in individuals with chronic diseases can also be effective in managing the disease [44, 45]. Dawson et al. (2021) found that reminder SMS messages sent to patients receiving HD treatment motivated patients to change their nutritional behaviors [46]. The literature reports that face-to-face education and reminder SMS messages administered to patients receiving HD treatment improved quality of life [47], improved treatment adherence, fluid restriction, and dietary adherence [48], and increased dialysis adherence [49]. In the present study, the combination of oral care education and reminders was associated with positive trends in oral health and chronic disease adherence. However, since the one-week follow-up period primarily reflects short-term behavioral responses rather than long-term habit formation, these results should be interpreted as an initial impact of the intervention. Literature on health behavior indicates that the formation of stable habits can take a significantly longer timeframe, with an average of 66 days required for a behavior to become automatic [50]. Therefore, these results should be interpreted as a promising initial impact of the educational intervention and reminder tools, while further studies are needed to evaluate long-term sustainability.

It is noteworthy that the rate of HD patients having previously received oral care education was low in this study. Addressing the neglect of oral care from the perspective of both the patient and the healthcare personnel can help control the factors affecting oral health. Furthermore, it is thought that maintaining interventions aimed at improving oral health may facilitate adherence to the disease. Furthermore, the findings of this study should be generalized with caution, as the research was conducted within two hospitals in a single geographic region. The specific healthcare environment and cultural context of the participants may influence oral health behaviors and adaptation levels differently than in other populations. When combined with the short-term intervention and follow-up period, these factors limit the external validity of the results across diverse healthcare systems and different cultural settings. Therefore, there is a need for multi-center studies that address the importance of oral health.

### Study limitations

This study has several limitations that should be considered when interpreting the findings. First, the use of surveys in the data collection instruments, and therefore its susceptibility to selection bias. A primary structural limitation is the single-group quasi-experimental design. The absence of a control group prevents the definitive attribution of improvements in oral health and illness adaptation scores solely to the educational intervention. Internal validity may have been influenced by the Hawthorne effect, where participants might have altered their behavior due to the awareness of being observed, or by the focused attention provided by the researchers during the intervention sessions. Consequently, the findings should be interpreted as short-term outcomes rather than definitive causal evidence.

Furthermore, the study’s generalizability is limited as it was conducted within two hospitals in a single geographic region. The specific healthcare environment and cultural context of the participants may influence oral health behaviors differently than in other populations. This, combined with the short follow-up period of one week, restricts the ability to assess the long-term sustainability of behavioral changes or the permanence of adaptation to chronic illness. To address these limitations, future research should utilize multi-center randomized controlled trials (RCTs) with longer follow-up periods to better isolate the effects of oral health education and evaluate its long-term clinical impact.

### Implications for practice

The findings emphasize the importance of addressing interventions for HD patients who are at risk in terms of oral health. Focusing on the reasons for patients’ neglect of oral care can prevent care-resistant behaviors. Evaluating the oral health of patients every time they come to the HD unit can raise awareness and facilitate their adherence to the disease. Oral health assessments should be added to the nursing care process. Oral health protocols should be implemented at regular intervals using standard forms, in line with the patients’ care requirements. Nurses working in the HD unit should be provided with training on oral health assessment, risk factors, and care protocols for these procedures. While this study suggests that the use of oral health reminder cards and checklists may help reduce the neglect of oral care in the short term, these findings should be interpreted with caution. Given the absence of a control group, it is recommended that clinical settings implement these tools as supportive awareness-raising measures rather than definitive preventive interventions. Future clinical practice should consider integrating these low-cost reminders into routine nursing care while further evaluating their sustained impact through controlled studies.

## Conclusions

In this study, it was observed that the oral health of HD patients was poor and their chronic disease adherence levels were moderate prior to the intervention. Baseline oral health was found to be influenced by income status and toothbrushing frequency, while chronic disease adherence levels varied according to education level, economic status, smoking status, and toothbrushing habits. Following the intervention, increased toothbrushing frequency emerged as a common factor associated with both improved oral health and higher chronic disease adherence. The implementation of the educational program, supported by reminder cards and checklists, was associated with positive short-term trends in these outcomes. However, these findings should be interpreted with caution. Due to the single-group design, the observed improvements may be influenced by the Hawthorne effect, research interest, or other external variables rather than the intervention alone. Therefore, the results provide preliminary evidence rather than definitive causal proof of the intervention’s effectiveness.

The study highlights that individuals with better oral health demonstrate higher chronic disease adherence. Addressing the underlying barriers that prevent HD patients from engaging in oral health-protective behaviors is crucial for comprehensive disease management. Consequently, there is a clear need for future multi-center studies that further investigate the impact of oral health on chronic disease outcomes. For clinical practice, the integration of low-cost tools, such as oral care reminders and checklists, is recommended to support patient self-management in dialysis settings.

## Supplementary Information

Supplementary Material 1.

## Data Availability

The datasets generated and/or analysed during the current study are not publicly available due privacy reasons but are available from the corresponding author on reasonable request.

## Abbreviations

ACIS: Adaptation to Chronic Illness Scale
HD: Hemodialysis
HSD: Tukey’s honestly significant difference
LSD: Fisher’s least significant difference
OAG: Oral Assessment Guide
r1: Pearson correlation analysis
r2: Spearman correlation
SD: standard deviation, value in bold: significant
T: The independent samples t-test
WHO: World Health Organization
