# Supplementary material for: The effect of oral care education and reminder cards on patients receiving hemodialysis treatment: a quasi-experimental study
Source: BMC Oral Health. 2026 Jul 15;26:1966. doi: 10.1186/s12903-026-09310-6 (PMC13651264; doi:10.1186/s12903-026-09310-6)
Supplement: Supplementary file 1 [file 12903_2026_9310_MOESM1_ESM.docx]

**DATA COLLECTION FORMS**

**Patient Information Form**

1. Age: ........... years
2. Gender: ( ) 1. Female ( ) 2. Male
3. Educational background: ( ) 1. Literate ( ) 2. Primary School ( ) 3. High School ( ) 4. Bachelor’s Degree ( ) 5. Graduate Degree
4. Marital Status: ( ) 1. Married ( ) 2. Single
5. Employment Status: ( ) 1. Employed ( ) 2. Unemployed ( ) 3. Retired
6. Income status:

( ) 1. Income less than expenses ( ) 2. Income equal to expenses ( ) 3. Income more than expenses

7. Person living together:

( ) 1. Living alone ( ) 2. Living with family (spouse, child, parents) ( ) 3. Living with a caregiver

8. Having a child: ( ) 1. Yes ( ) 2. No
9. Smoking status: ( ) 1. Yes ( ) 2. No
10. Alcohol use status: ( ) 1. Yes ( ) 2. No
11. Duration of hemodialysis treatment: ……………. months/years
12. Presence of chronic diseases other than Chronic Kidney Disease: ( ) 1. Yes ( ) 2. No
13. Total number of medications used: ...........
14. Oxygen therapy: ( ) 1. Yes ( ) 2. No
15. Status of receiving oral care training: ( ) 1. Yes ( ) 2. No
16. Daily tooth brushing status: ...........
17. Tooth brushing status after training: ( ) 1. Yes ( ) 2. No

**Oral Health Reminder Card**

**Oral Health Checklist**

|  |  | ......  day | ......  day | ......  day | ......  day | ......  day | ......  day | ......  day |
| --- | --- | --- | --- | --- | --- | --- | --- | --- |
| 1 | Brush your teeth regularly to protect your oral and dental health. |  |  |  |  |  |  |  |
| 2 | Brush your teeth daily at the frequency your nurse advised you. |  |  |  |  |  |  |  |
| 3 | When performing your oral care, treat your mouth as a whole and clean your tongue, palate, and between your teeth. |  |  |  |  |  |  |  |
| 4 | Avoid brushing your teeth harshly/aggressively. |  |  |  |  |  |  |  |
| 5 | Use dental floss and mouthwashes that will contribute to your oral health. |  |  |  |  |  |  |  |
| 6 | Ensure your fluid intake every 2 hours as per your doctor's recommendation. |  |  |  |  |  |  |  |
| 7 | Apply moisturizing cream to your lips every 2 hours and/or as needed. |  |  |  |  |  |  |  |
| 8 | Avoid spicy foods that may irritate the inside of your mouth. |  |  |  |  |  |  |  |
| 9 | Avoid consuming very hot and very cold drinks and foods. |  |  |  |  |  |  |  |
| 10 | Brush your teeth after consuming items that cause staining on the teeth. |  |  |  |  |  |  |  |
